# FV-429 induces autophagy blockage and lysosome-dependent cell death of T-cell malignancies via lysosomal dysregulation

**DOI:** 10.1038/s41419-021-03394-4

**Published:** 2021-01-13

**Authors:** Po Hu, Jubo Wang, Yingjie Qing, Hui Li, Wenzhuo Sun, Xiaoxuan Yu, Hui Hui, Qinglong Guo, Jingyan Xu

**Affiliations:** 1 State Key Laboratory of Natural Medicines, Jiangsu Key Laboratory of Carcinogenesis and Intervention, China Pharmaceutical University, 24 Tongjiaxiang, Nanjing, 210009 China; 2 Jiangsu Key Laboratory of Drug Design and Optimization, Department of Medicinal Chemistry, School of Pharmacy, China Pharmaceutical University, Nanjing, 210009 China; 3 Department of Pharmacology, School of medicine & Holostic integrative medicine, Nanjing University of Chinese Medicine, Nanjing, 210046 China; 4 Department of Hematology, The Affiliated DrumTower Hospital of Nanjing University Medical School, Nanjing, 210008 China

**Keywords:** Haematological cancer, Macroautophagy, Pharmacology

## Abstract

It is widely accepted that lysosomes are essential for cell homeostasis, and autophagy plays an important role in tumor development. Here, we found FV-429, a synthetic flavonoid compound, inhibited autophagy flux, promoted autophagosomes accumulation, and inhibited lysosomal degradation in T-cell malignancies. These effects were likely to be achieved by lysosomal dysregulation. The destructive effects of FV-429 on lysosomes resulted in blockage of lysosome-associated membrane fusion, lysosomal membrane permeabilization (LMP), and cathepsin-mediated caspase-independent cell death (CICD). Moreover, we initially investigated the effects of autophagy inhibition by FV-429 on the therapeutic efficacy of chemotherapy and found that FV-429 sensitized cancer cells to chemotherapy agents. Our findings suggest that FV-429 could be a potential novel autophagy inhibitor with notable antitumor efficacy as a single agent.

## Introduction

Macroautophagy (herein after referred to as autophagy) is a lysosome-dependent degradation process of damaged or unnecessary cellular components^1,2^. The autophagic substrates including damaged organelles and protein aggregates are engulfed by double-membrane autophagosomes and fused with lysosomes for degradation^2^. It has been proved that autophagy plays an important role in the survival of cancer cells, or contribute to cell death^3^. However, the mechanism of autophagy regulation in tumors is still not clear^3,4^. Although the cell demise induced by autophagy is recognized, in general, the cancer cells tend to activate autophagy for self-rescue and maintaining homeostasis under stress condition^5–7^. Increasing studies have implicated that the role of protective autophagy participates in the development of multidrug resistance and protects cancer cells from chemotherapeutics or radiotherapy, suggesting that targeting the protective autophagy process is regarded as a novel therapeutic approach^8,9^.

Lysosomes, the acidic compartments, play a role in degradation at the terminal step of autophagy^2^. The lysosomal dysfunction may induce autophagy blockage by inhibiting endocytic trafficking or substrate degradation^10^. Tumor cells rely more on increased lysosomal function to proliferate and for metabolism, and are sensitive to lysosomal dysregulation^11,12^. Lysosome-dependent cell death (LCD) is mainly caused by lysosomal membrane permeabilization (LMP), a process in which the lysosome loses its membrane integrity and lysosomal contents are released into the cytosol^13^. LMP leads to caspase-dependent or caspase-independent cell death (CICD), with emergence of lysosomes as promising therapeutic targets for several human diseases^14,15^.

T-cell malignancies encompass a heterogeneous group of diseases, each reflecting a clonal evolution of dysfunctional T cells at various stages of development^16^. It encompasses immature (i.e., T-cell acute lymphoblastic leukemias (T-ALL)) and mature (i.e., T cell lymphomas (TCL)) lymphoid neoplasms and are often associated with a dismal prognosis^17^. The current treatment strategies for T-cell malignancies, such as chemotherapies, radiation, and hematopoietic stem cell transplantation, are often accompanied with relapse, drug resistance, and toxicities^18^. It is reported that autophagy plays important roles in metabolism, differentiation, and survival in T cells, as well as in hematological malignancy development and drug resistance^19,20^. Additionally, studies have found that treatment of T-cell malignancies is accompanied with autophagy regulation^21–24^. Emerging reports have indicated that some flavonoids promote cell death by mediating autophagy in tumor cells^25–27^, such as wogonin^28–30^. FV-429, a derivative of wogonin, has been proved to exert anti-cancer effects in several solid tumors^31–33^. However, the exact mechanism by which FV-429 regulates autophagy remains unclear. Here, we attempt to demonstrate the effects of FV-429 on autophagy in T-cell malignancies, as well as to explore its activity and mechanism of antitumor effects.

## Materials and methods

### Cell culture and reagents

Human T-ALL cell lines (Jurkat, Molt4), TCL cell lines (Hut102), and human embryonic kidney cells (293 T) were purchased from Cell Bank of Shanghai Institute of Biochemistry & Cell Biology. Peripheral blood mononuclear cells (PBMCs) derived from healthy donor and patients with newly diagnosed T-cell malignancies without prior therapy (The First Affiliated Hospital of Nanjing Medical University, Nanjing, China) were collected using lymphocyte-monocyte separation medium (Jingmei, Nanjing, China). Primary cells #1 belongs to anaplastic large cell lymphoma (ALCL), a lymphoma of mature peripheral T cells. Primary cells #2 belongs to immature T-ALL. The suspended cells were cultured in RPMI-1640 medium (GIBCO, USA) and 293 T cells were cultured in DMEM medium (GIBCO, USA). The mediums were supplemented with 10% fetal bovine serum (FBS) (GIBCO, USA), 100 U/mL of benzylpenicillin, and 100 U/mL of streptomycin in a humidified environment with 5% CO_2_ at 37 °C. The identity of each cell line was confirmed by short tandem repeat (STR) profiling. All cell lines were regularly tested for Mycoplasma spp. infection with DAPI staining. FV-429 was synthesized and provided by Prof. Zhiyu Li in our lab. Bafilomycin A1 (BAF A1) (KGATGR007) and Lysotracker RED (KGMP006) were purchased from KeyGene (China). Acridine orange (AO-HY-101879) was purchased from MCE (China). Necrostatin-1 (Nec-1) (CSN11637), Z-VAD-fmk (CSN15936), BAPTA-AM (CSN10377), and Rapamycin (CSN16385) were purchased from CSNpharm (USA). Lysosensor GREEN (40767ES50) and Cell Counting Kit (CCK8, 40203ES60) were purchased from YEASEN (China). Antifade mounting medium (P0126) and antifade mounting medium with DAPI (P0131) were purchased from Beyotime Biotechnology (China). Antibodies for caspase 3 (A2156), caspase 9 (A0281), caspase 8 (A0215), β-actin (AC026), microtubule-associated protein light chain 3 (LC3) (A17424), and sequestosome 1 (p62/SQSTM1) (A19700) were obtained from ABclonal Technology (Wuhan, China). Lysosomal membrane protein 1 (LAMP1) (15665) was purchased from Cell Signaling Technology. Ubiquitin (10201-2-AP), RAB7A (55469-1-AP), and LC3 (14600-1-AP) were purchased from Proteintech Group. The secondary antibodies HRP Goat Anti-Rabbit IgG (H + L) (AS014) and HRP Goat Anti-Mouse IgG (H + L) (AS003) were obtained from ABclonal Technology, and Goat Anti-Rabbit/Mouse IgG (H + L) (A-11008, A-11001, R37117, and A-11032) was purchased from ThermoFisher Scientific (USA).

### Western blot

Whole-cell lysates were extracted from cells after treatment with RIPA buffer (ThermoFisher Scientific, 89901). The concentration of protein was determined by BCA Protein Assay Kit (ThermoFisher Scientific, 23227). Equal amounts of lysate protein were resolved on SDS-PAGE gels and transferred to NC membrane followed by western blot analysis. Immunodetection was performed using an enhanced chemiluminescence ECL system (Yeason, 36222ES60). Detection was performed with Amersham Imager 600 (General Electric Company, USA). The blots are representative of multiple independent experiments.

### Transfections and RNA interference

Transfections were achieved using LipoMAX (SUDGEN, 32011) according to the manufacturer’s protocol. Cells were transfected with 3 μL LipoMAX and 3 μg plasmids encoding Galectin-3-mcherry (#85662), LC3-GFP (#11546), and LC3-GFP-mcherry (#123235) from Addgene in 500 μL serum-free medium. After 72 h incubation, the transfection mixture was removed and replaced with fresh complete medium. For RNA interference by lentiviral vectors, *cathepsin D* (*CTSD*) short hairpin RNA (shRNA), *cathepsin B* (*CTSB*) shRNA constructs, and a negative control construct created in the same vector system (pLKO.1) were purchased from Corues Biotechnology. Before transfection, 293 T cells were plated in 12-well dishes. Cells were co-transfected with shRNA constructs (10 μg) together with Lentiviral Mix (10 μL) and HG Transgene^TM^ Reagent (60 μL) according to the manufacturer’s instructions of Lentiviral Packaging Kit (YEASEN, 41102ES20) for 2 days, viral stocks were harvested from the culture medium and filtered to remove non-adherent 293 T cells. To select the Jurkat cells that were stably expressing shRNA constructs, cells were incubated in RPMI-1640 medium with 10% FBS and 2 μg/mL of puromycin for 48 h after lentivirus infection. After selecting cells, stably infected pooled clones were harvested for use.

### Immunofluorescence

Cells were collected and washed with phosphate-buffered saline (PBS) twice and smeared on the cover glass. Then the cells were fixed in ice-cold methanol for 15 min and permeabilized in 0.15% Triton X-100 for 20 min. After blocking with 3% BSA for 1 h at room temperature, the cells were incubated with primary antibody overnight at 4 °C, followed by incubation with Alexa Fluor secondary antibody for 1 h and DAPI. The cells were observed with a confocal laser scanning microscope (Fluoview FV1000, Olympus, Tokyo, Japan). The images were analyzed by Image-Pro Plus 6.0 software (cell counts >100).

### AO staining

Cells were stained with 5 μM AO solution in culture medium for 15 min before collecting. Cells were washed with PBS twice and smeared on the cover glass. Then the cells were observed with a confocal laser scanning microscope.

### Lysotracker RED and lysosensor GREEN staining

The cells stained with 0.5 μM lysotracker RED for 45 min in cell culture medium at 37 °C were analyzed for the lysosomal mass. For analyzing the lysosomal pH, the cells were stained with 1 μM lysosensor GREEN for 20 min in cell culture medium at 37 °C. After washing with PBS, the processed cells were suspended and analyzed by flow cytometry or detected by fluorescence microscope.

### CCK8 assay

PBMCs and cell lines seeded in 96-well plates were treated with FV-429, then, 10 μL CCK8 solution was added to each well and incubated for 4 h at 37 °C. Cells treated with equivalent amounts of DMSO were negative control. Absorbance was read at 450 nm with a SynergyTM HT multi-mode reader (Bio-Tek, Winoosky, VT). The average value of the optical density (OD) of five wells was used to determine cell viability according to the following formula: Inhibition rates(%) = (1−OD_treatment group_/OD_control group_) × 100%. IC_50_ values were taken as the concentration that caused 50% inhibition of cell viability and were calculated by the *logit* method.

### Cell death detection

The cells were collected and labeled with Annexin V and PI according to the protocols of Annexin V/PI Cell Apoptosis Detection Kit (Vazyme biotec, A211-02)^34^. The dead cells were Annexin V-positive. The fluorescence was detected by Becton-Dickinson FACSCalibur flow cytometry and the data analysis was performed by FlowJo software.

### Mitochondrial membrane potential

After treatment the cells were harvested and processed with JC-1 (Beyotime Biotechnology, C2006) according to the manufacturer’s instructions^34^. Then, processed cells were analyzed by flow cytometry.

### Statistical analysis

All data were expressed as mean ± s.e.m. from 3 independent experiments performed in a parallel manner. Statistical analysis of multiple group comparisons was performed by one-way analysis of variance (ANOVA) followed by the Bonferroni post hoc test. Comparisons between two groups were analyzed using two-tailed Student’s *t*-tests. The *p* values were indicated on the graph and *p* < 0.05 was considered statistically significant.

## Results

### FV-429 promoted autophagy vesicles accumulation

**Fig. 1:**
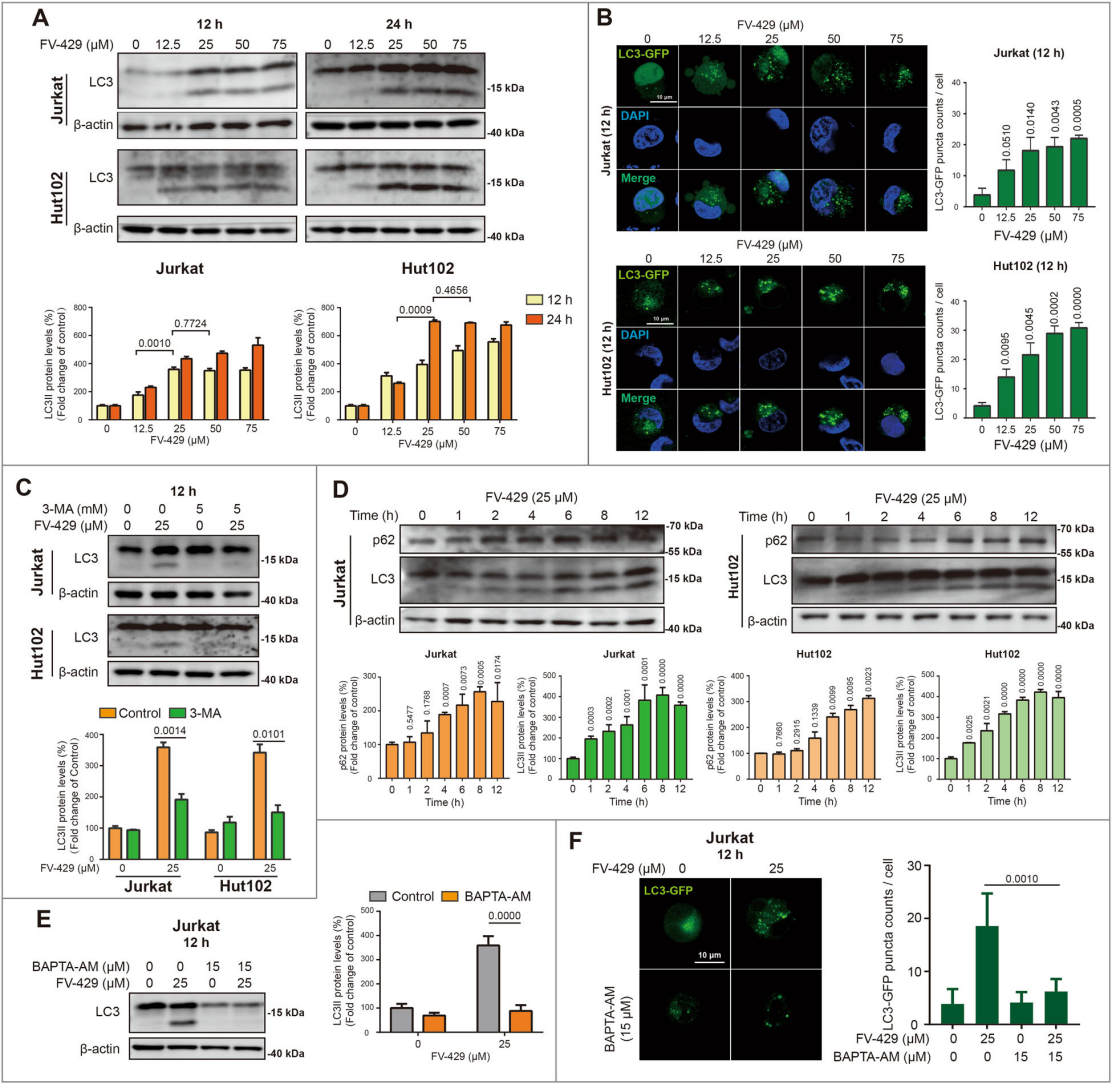
FV-429 promotes LC3II accumulation. **A** The Jurkat and Hut102 cells were treated with 0, 12.5, 25, 50, and 75 μM FV-429 for 12 and 24 h. The expression of LC3 was determined by western blot. **B** The Jurkat and Hut102 cells were transfected with a plasmid encoding LC3-GFP and treated with 0, 12.5, 25, 50, and 75 μM for 12 h. The LC3II puncta determined by fluorescence microscopy (scale bar: 10 μm; the cells calculated in each group >50). **C** The Jurkat and Hut102 cells were treated with 25 μM FV-429 and 3-MA (5 mM, pre-treated for 2 h) for 12 h. The protein expression of LC3 was determined by western blot. **D** The Jurkat and Hut102 cells were treated with 25 μM FV-429 for 0, 1, 2, 4, 6, 8, and 12 h. The protein expression of LC3 and p62 was determined by western blot. **E** The Jurkat cells were treated with 25 μM FV-429 and BAPTA-AM (15 μM, pre-treated for 1 h) for 12 h, the protein expression of LC3 was determined by western blot. β-actin used as loading control in western blot. **F** The Jurkat cells were transfected with a plasmid encoding LC3-GFP and treated with 25 μM FV-429 and BAPTA-AM (15 μM, pre-treated for 1 h) for 12 h. The LC3II puncta determined by fluorescence microscopy, and LC3II-GFP puncta count were calculated (scale bar: 10 μm; total puncta in each group >200) (mean ± s.e.m. for 3 independent experiments; *p* values are shown on the graph).

To study the effects of FV-429 on autophagic pathway in human T-cell malignancies, firstly, the expression of autophagy marker protein LC3 was determined by western blot. LC3II expression increased in Jurkat and Hut102 cells incubated with 0~75 μM FV-429 for 12 and 24 h (Fig. 1A). In Jurkat cells treated with FV-429 for 12 and 24 h, or Hut102 cells treated with FV-429 for 24 h, the expressions of LC3II increased to plateau at high concentrations of FV-429 (25~75 μM). It is suggested that FV-429 promoted accumulation of autophagy vesicles, and that effect reached saturation point for the treatment with 25 μM FV-429. Autophagy vesicle accumulation was also confirmed in cells expressing LC3-GFP transiently. LC3-GFP puncta increased after 12.5~75 μM FV-429 treatment for 12 h in cells transfected with LC3-GFP plasmid (Fig. 1B). The expression of LC3II induced by FV-429 was inhibited by 3-methyladenine (3-MA) (Fig. 1C), an early-stage autophagy inhibitor. Western blot analysis also showed that treatment with 25 μM FV-429 resulted in time-dependent promotion on LC3II expression during 0~12 h, as well as on sequestosome 1, p62/SQSTM1 (p62) expression (Figs. 1D and S1A).

In addition, FV-429 induced autophagy vesicle formation in calcium-dependent manner. In Jurkat and Hut102 cells, results showed that cytoplasmic calcium chelator (BAPTA-AM) decreased FV-429-induced LC3II expression markedly (Figs. 1E and S1B). Treatment with BAPTA-AM counteracted FV-429-evoked increase of LC3-GFP puncta count (Figs. 1F and S1C). In contrast, in Molt4 cells, BAPTA-AM promoted the FV-429-induced expression of LC3II (Fig. S1D). These data initially proved that calcium regulated FV-429 induced autophagy vesicle accumulation, specific to certain types of cells.

### FV-429 blocked autophagy flux and autophagic degradation

**Fig. 2:**
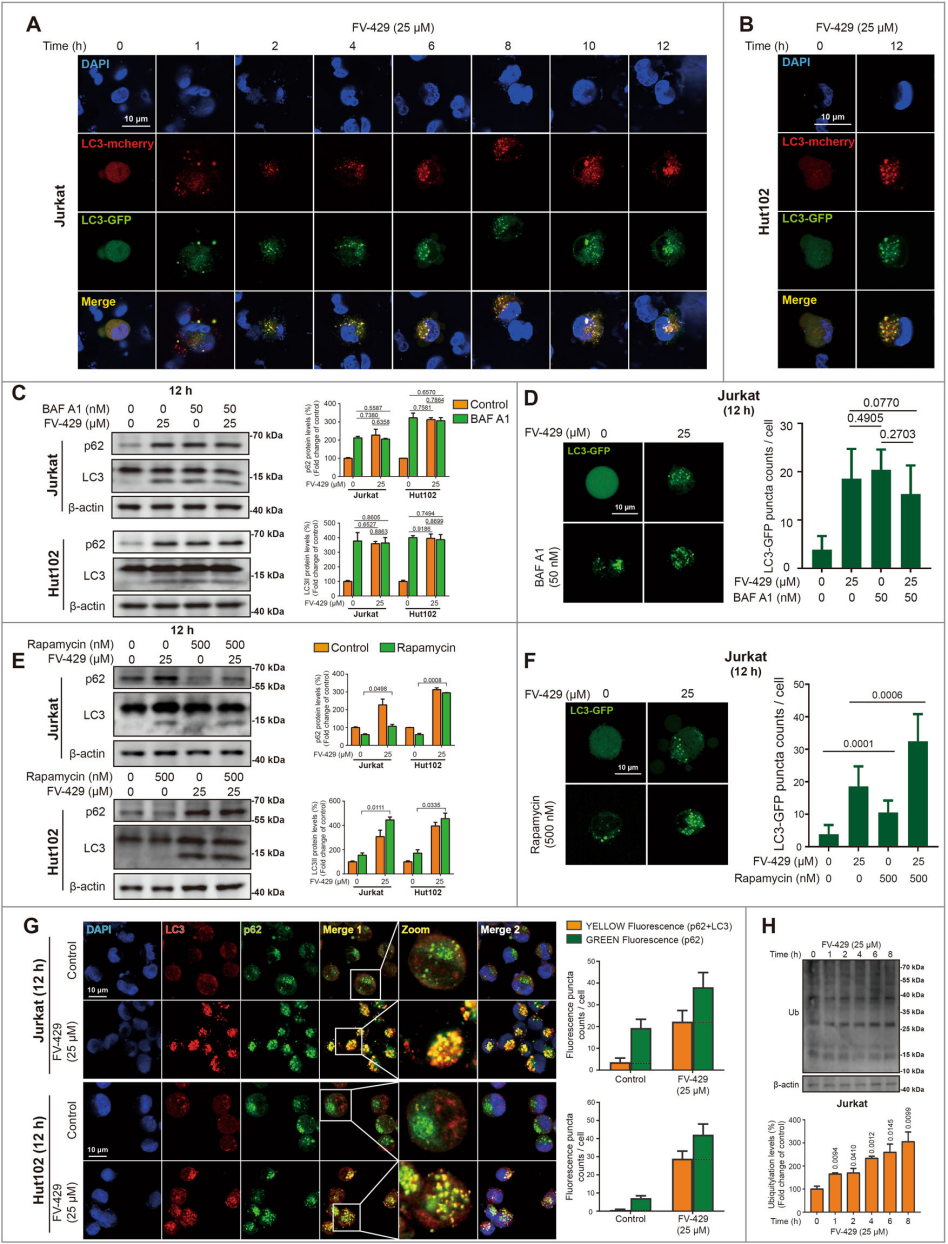
FV-429 inhibits autophagy flux and induces degradative substrate accumulation. **A, B** The cells transfected with a plasmid encoding LC3-GFP-mcherry were incubated with 25 μM FV-429 for 0~12 h in Jurkat and for 12 h in Hut102 cells. The LC3II puncta formation was detected by fluorescence microscopy (scale bar: 10 μm). **C** The Jurkat and Hut102 cells were treated with 25 μM FV-429 and BAF A1 (50 nM, pre-treated for 1 h) for 12 h, and the protein expression of LC3 and p62 was determined by western blot. **D** The Jurkat cells were transfected with a plasmid encoding LC3-GFP and treated with 25 μM FV-429 and BAF A1 (50 nM, pre-treated for 1 h) for 12 h. The LC3II puncta determined by fluorescence microscopy, and LC3II-GFP puncta count were calculated (scale bar: 10 μm; total puncta in each group >200). **E** The Jurkat and Hut102 cells were treated with 25 μM FV-429 and rapamycin (500 nM, pre-treated for 2 h) for 12 h, and the protein expression of LC3 and p62 was determined by western blot. **F** The Jurkat cells were transfected with a plasmid encoding LC3-GFP and treated with 25 μM FV-429 and rapamycin (500 nM, pre-treated for 2 h) for 12 h. The LC3II puncta determined by fluorescence microscopy, and LC3II-GFP puncta count were calculated (scale bar: 10 μm; total puncta in each group >200). **G** The Jurkat and Hut102 cells were treated with 25 μM FV-429 for 12 h. The immunofluorescence analysis was performed with anti-p62 antibody (green), anti-LC3 antibody (red; autophagosomes), and DAPI (blue; nuclei). The counts of p62 puncta and co-localization of p62 and LC3 were calculated (scale bar: 10 μm, the cells calculated in each group >100). **H** The Jurkat cells were treated with 25 μM FV-429 for 0~8 h and the expressions of ubiquitin were determined by western blot. β-actin used as loading control in western blot (mean ± s.e.m. for 3 independent experiments; *p* values are shown on the graph).

p62 links LC3 and substrates destined for degradation. Upon autophagy, p62 is degraded with a cargo, thus elevated level of p62 is correlated with autophagy inhibition^2^. In order to determine the effects of FV-429 on autophagy flux, the cells were transfected with LC3-GFP-mcherry, which can reflect progress conditions of autophagy flux^2,35^. In Jurkat cells, exposure to 25 μM FV-429 caused significant formation of LC3II-GFP at the timepoint of 1 h, overlaying with LC3II-mcherry completely and displaying yellow fluorescence during 2~12 h (Fig. 2A). In Hut102 and Molt4 cells, exposure to FV-429 for 12 h also displayed the puncta formation with yellow overlay (Figs. 2B and S2A). It is demonstrated that autophagy flux was inhibited by FV-429. Additionally, we used Bafilomycin A1 (BAF A1), a vacuolar-type ATPase inhibitor^2^, to assess whether treatment with FV-429 altered complete autophagic flux by determining the expression of LC3II. Compared with cells treated with 25 μM FV-429, LC3II protein expressions were not enhanced in the cells co-treated with BAF A1 and FV-429, or BAF A1 alone (Fig. 2C). LC3II expression levels of FV-429 group, BAF A1 group, and combination group were almost equal, which was also verified by LC3-GFP detection (Figs. 2D and S2B). The results above suggested that FV-429 had a similar effect to BAF A1 on autophagy flux. Therefore, 25 μM FV-429-induced accumulation of LC3II was due to autophagy inhibition at late stage.

Until the degradative substrates transfer to lysosomes for degradation completely, the intact autophagy is finished^2^. We further confirmed the lysosomal degradative inhibition of FV-429. Rapamycin can induce autophagy activation via mTOR inhibition^36^. In Jurkat and Hut102 cells, compared with rapamycin-treated alone, the combination of rapamycin and FV-429 enhanced the expression of LC3II and p62 protein (Fig. 2E). Combination of rapamycin and FV-429 also promoted LC3II-GFP puncta formation in both the cells transfected with LC3-GFP (Figs. 2F and S2C). It was suggested that FV-429 blocked the autophagy flux activated by rapamycin. Next, FV-429 treatment resulted in accumulation of p62 puncta and increased co-localization with LC3II puncta (Fig. 2G). Compared with control group, the co-localization of p62 and LC3II increased from 15.97 ± 8.45% to 57.21 ± 4.98% in Jurkat cells, and 7.83 ± 6.78% to 68.43 ± 1.42% in Hut102 cells, respectively. Thus, it could be concluded that the degradation of lysosomal substrates linked with p62 and co-localized with LC3II was inhibited. Furthermore, FV-429 increased the amount of ubiquitinated proteins in the cells, which also suggested the substrate degradation inhibition (Fig. 2H). Taken together, FV-429 inhibited autophagy flux, thereby resulting in accumulation of autophagic vesicles.

### FV-429 inhibited lysosome-associated membrane fusion

**Fig. 3:**
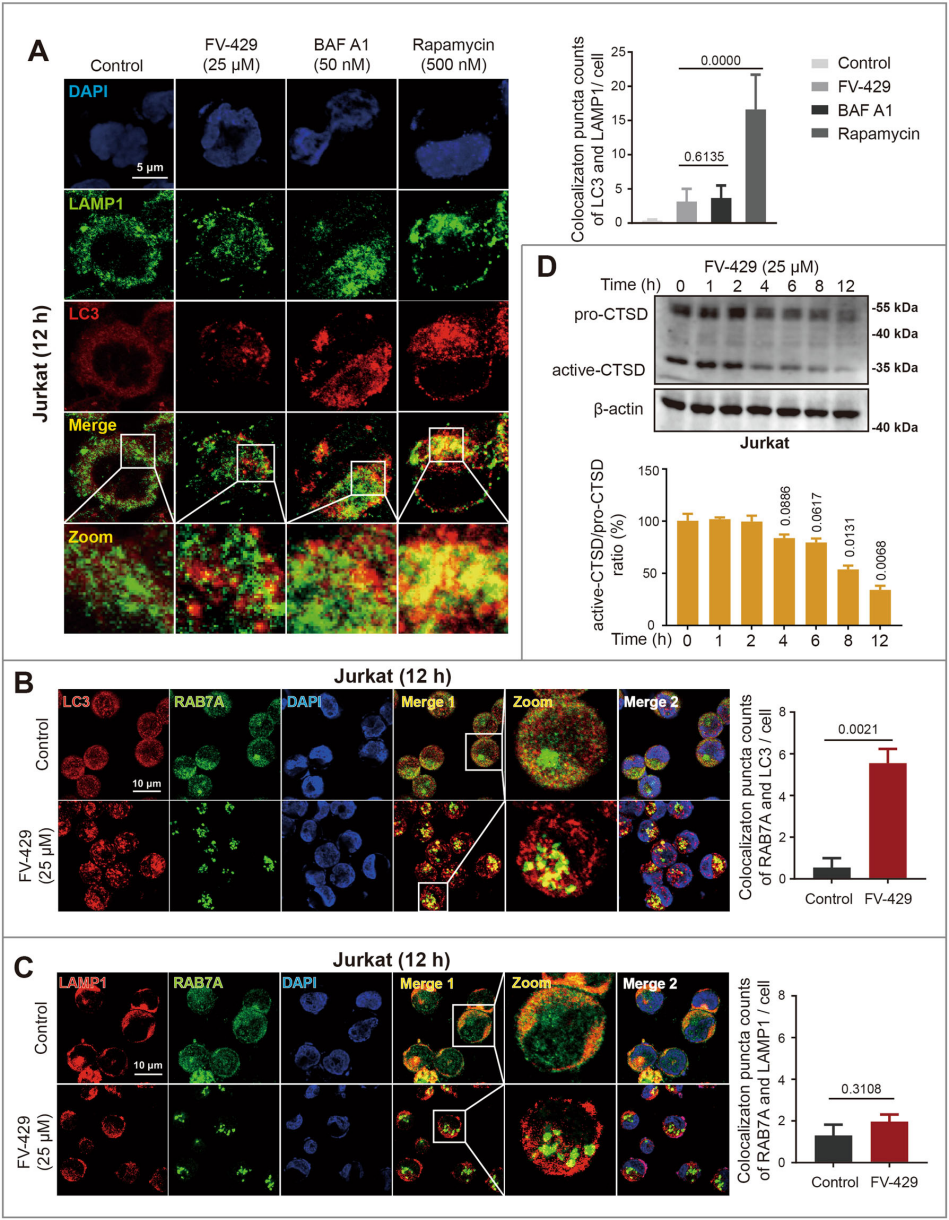
FV-429 blocks lysosome-associated membrane fusion. **A** The Jurkat cells were treated with FV-429, BAF A1, and rapamycin for 12 h. The immunofluorescence analysis was performed with anti-LAMP1 antibody (green; lysosomes), anti-LC3 antibody (red; autophagosomes), and DAPI (blue; nuclei). The overlay levels were analyzed (scale bar: 5 μm, the cells calculated in each group >100). **B**, **C** The Jurkat cells were treated with 25 μM FV-429 for 12 h. The immunofluorescence analysis was performed with anti-LC3 antibody (red; autophagosomes), anti-RAB7A antibody (green), and DAPI (blue; nuclei) (**B**); or performed with anti-LAMP1 antibody (red; lysosomes), anti-RAB7A antibody (green), and DAPI (blue; nuclei) (**C**). The co-localization counts of RAB7A with LC3 or LAMP1 were calculated (scale bar: 10 μm, the cells calculated in each group >100). **D** The Jurkat cells were treated with 25 μM FV-429 for 0~12 h, and the expression of CTSD was determined by western blot. β-actin used as loading control (mean ± s.e.m. for 3 independent experiments; *p* values are shown on the graph).

The autophagosome or autolysosome accumulation might result from the autophagy failure at the fusion or degradation phase, respectively^2^. Previous studies have illustrated that autophagosome-lysosome fusion is promoted by rapamycin, while is inhibited by BAF A1^35,37^. To address whether FV-429 impaired autophagosome-lysosome fusion, first, we used confocal microscopy to assess the co-localization of LC3 with lysosome-associated membrane protein 1 (LAMP1). Notably, LC3II puncta increased in Jurkat cells on treatment with 25 μM FV-429, but were not co-localized with LAMP1 (Fig. 3A). This analysis was also performed in Hut102 and Molt4 cells (Fig. S3A). The findings indicated that FV-429 blocked autophagosome-lysosome fusion. RAB7A is required for the fusion process of multiple membranes including lysosomes and endosomes/autophagosomes^38^. It is revealed that the 25 μM FV-429 promoted the aggregation of RAB7A and its co-localization with LC3 (Figs. 3B and S3B). In contrast, co-localization of RAB7A and LAMP1 was not affected by FV-429 (Figs. 3C and S3C). It is suggested that FV-429 treatment inhibited the recruitment of RAB7A on lysosomes, which could result in autophagosome-lysosome fusion failure.

It is prompted that FV-429 might cause the lysosomal disturbance based on the phenomenon of inhibitory effects of FV-429 on recruitment of RAB7A to lysosomes instead of autophagosomes. Therefore, we further determined endosome-lysosome fusion to initially confirm whether FV-429 induced lysosomal abnormity. The maturation of cathepsins is dependent on endocytic pathway for transport into the lysosomes, and the active form of cathepsin expression reflected the progression of endosome-lysosome fusion^15^. Both in Jurkat and Hut102 cells treated with FV-429, the active- CTSD/pro-CTSD ratio decreased significantly, suggesting the maturation of CTSD was inhibited (Figs. 3D and S3D). These results proved that the FV-429-induced lysosome-associated membrane blockage might be triggered by lysosomal abnormity.

### FV-429-induced lysosomal damage and lysosomal membrane permeabilization

**Fig. 4:**
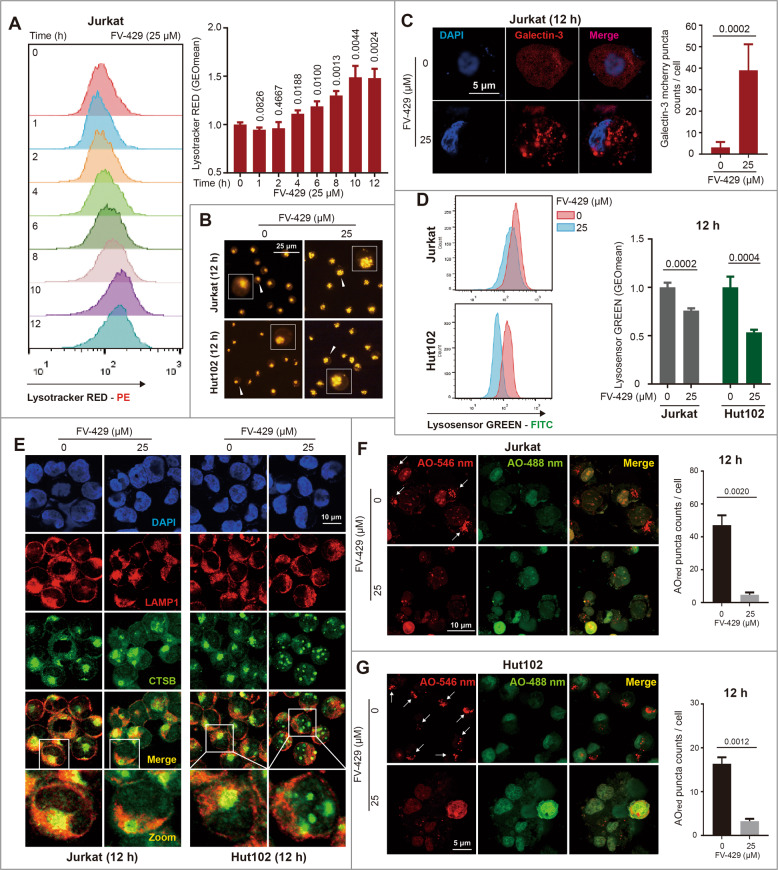
FV-429 induces lysosomal damage and LMP. **A** The Jurkat cells treated with 25 μM FV-429 for 0~12 h were stained by Lysotracker RED and detected by flow cytometry. GEOmean of fluorescence intensity was analyzed by FlowJo software. **B** The Jurkat and Hut102 cells were treated with 25 μM FV-429 for 12 h and stained with Lysotracker RED. The morphology was determined by fluorescent microscope (scale bar: 25 μm). **C** The Jurkat cells transfected with Galectin-3 mcherry plasmid were treated with 25 μM FV-429 for 12 h and the Galectin-3 puncta were calculated (scale bar: 5 μm; total puncta in each group >200). **D** The Jurkat and Hut102 cells treated with 25 μM FV-429 for 12 h were stained with Lysosensor GREEN and detected by flow cytometry to determine the lysosomal pH. **E** The Jurkat and Hut102 cells were treated with 25 μM FV-429 for 12 h. The immunofluorescence analysis was performed with anti-LAMP1 antibody (red; lysosomes), anti-CTSB antibody (green), and DAPI (blue; nuclei) (scale bar: 10 μm). **F**, **G** The Jurkat and Hut102 cells treated with 25 μM FV-429 for 12 h were stained with AO staining before collecting, and the AO fluorescences were determined by laser confocal microscope (the scale bars in Jurkat group and Hut102 group are 10 and 5 μm, respectively). The AO_red_ puncta count per cell (*n* > 50) were calculated (mean ± s.e.m. for 3 independent experiments; *p* values are shown on the graph).

Lysosomal disorder and the insufficient degradation function induce autophagic flux inhibition, lysosomal substrate accumulation, and even cause lysosomal storage disorder diseases^39^. Lysotracker RED is a lysosomal probe with a red fluorescence emission^40^. It was used to determine the morphology of lysosomes. In Jurkat, Hut102, and Molt4 cells, 25 μM FV-429 increased the fluorescence intensity of lysotracker RED during 0~12 h (Figs. 4A and S4A) in a time-dependent manner, accompanied with the enlargement of lysosome volume (Figs. 4B and S4B). The lysosomal dysregulation was also confirmed by the detection of Galectin-3, which is distributed throughout the cytoplasm and nuclei, is recruited to injured lysosomes and forms Galectin-3 puncta^41^. As shown in Figs. 4C and S4C, the Galectin-3 puncta formation was increased after FV-429 treatment in the cells transfected with Galectin-3-mcherry plasmid, indicating an abnormal lysosome enlargement and damage.

LMP is a typical phenomenon of lysosomal alterations, which is accompanied with leakage of lysosomal content, including proton and hydrolases^42,43^. Due to FV-429-induced injured lysosomes, we confirmed whether FV-429 triggered LMP. Firstly, Lysosensor GREEN assay revealed a higher pH of acidic organelles after FV-429 treatment (Fig. 4D). Next, as shown in Figs. 4E and S4D, the increased CTSB protein levels in cytoplasm induced by FV-429 revealed the lysosome membrane barriers were broken by determining the co-localization of CTSB and LAMP1. Besides, LMP can be assessed by AO method, which is used to detect acid vesicles and emit red fluorescence^44^. AO-labeled vesicles decreased significantly in Jurkat and Hut102 cells treated with FV-429 (Fig. 4F, G), indicating the loss of acidic environment in lysosomes. These data suggested that FV-429 had disruptive effects on lysosomes, triggering lysosome damage and promoting the leakage of lysosomal contents.

### FV-429 promoted cathepsin-mediated caspase-independent cell death

**Fig. 5:**
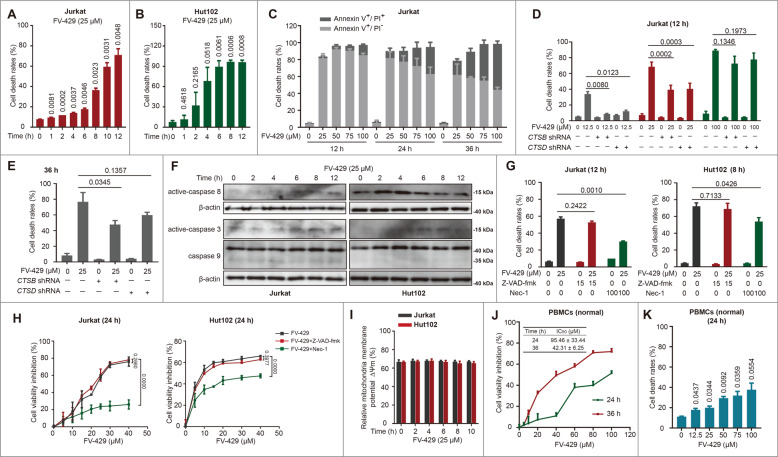
FV-429 induces cathepsin-associated necroptosis. **A**, **B** The Jurkat and Hut102 cells were treated with 25 μM FV-429 for 0~12 h. **C** The Jurkat cells were treated with 0, 25, 50, 75, and 100 μM FV-429 for 12, 24, and 36 h. **D**, **E** The Jurkat cells transfected with *CTSB* and *CTSD* shRNA were treated with 12.5, 25, or 100 μM FV-429 for 12 h (**D**) or 36 h (**E**). **F** The Jurkat and Hut102 cells were treated with 25 μM FV-429 for 0~12 h, and the expressions of caspase 3, 8, and 9 were determined by western blot. β-actin used as loading control. **G** The cells were pre-treated with Z-VAD-fmk (15 μM) or Nec-1 (100 μM) for 2 h and then incubated with 25 μM FV-429 for 12 h in Jurkat and 8 h in Hut102 cells. The cells above were stained with Annexin V/PI staining and cell death rates were detected by flow cytometry (dead cells are positive for Annexin V). **H** The Jurkat and Hut102 cells were pre-treated with Z-VAD-fmk (15 μM) or Nec-1 (100 μM) for 2 h and then incubated with 0~40 μM FV-429 for 24 h. The cell viability inhibition was determined by CCK8 assay. **I** The Jurkat and Hut102 cells were treated with 25 μM FV-429 for 0~10 h, and the mitochondria membrane potentials (∆Ψm) were determined by JC-1 assay. Relative ∆Ψm was calculated by taking the ratio of red fluorescence in treated cells with respect to controls. **J** The PBMCs derived from healthy donor were collected from health volunteer, and treated with 0~100 μM FV-429 for 24 and 36 h. The cell viability inhibition was determined by CCK8 assay. **K** The PBMCs were derived from healthy donor, and treated with 0~100 μM FV-429 for 24 h. The cells were stained with Annexin V/PI staining and cell death rate was detected by flow cytometry (dead cells are positive for Annexin V) (mean ± s.e.m. for 3 independent experiments; *p* values are shown on the graph).

The cytotoxicity of FV-429 was also determined in cells of T-cell malignancies. Treating the cells with 25 μM FV-429 promoted the cell death rates significantly in a time-dependent manner (Figs. 5A, B and S5A). At the timepoint of 12 h, the cell death rate reached 71.01 ± 4.41%, 96.22 ± 1.99%, and 78.48 ± 4.24% in Jurkat, Hut102, and Molt4 cells, respectively. Also, 25~100 μM FV-429 increased cell death of Jurkat cells for the duration of 12~36 h, and results showed that rates for Annexin V-positive and PI-positive increased in a time- and concentration-dependent manner (Fig. 5C). Next, we explore the initial factors of FV-429-induced cell death. Previous studies have shown that the leakage of cathepsins play a major role in LCD^15^. We assessed the cell death rates in Jurkat cells transfected with *CTSB* or *CTSD* shRNA. The results showed that both of *CTSB* and *CTSD* shRNA reduced 12.5 μM and 25 μM FV-429-induced cell death rates, respectively, notably at 12 h (Fig. 5D). *CTSB* shRNA also inhibited 25 μM FV-429-induced cell deaths at 36 h (Fig. 5E). It is demonstrated that CTSB and CTSD were involved in FV-429-induced cell death.

Furthermore, we determined the activation of caspase family protein after FV-429 treatment. FV-429 slightly increased cleaved-caspase 3 and had no effect on cleaved-caspase 9 expressions in Jurkat and Hut102 cells (Fig. 5F). As for cleaved-caspase 8, its expression was increased slightly by FV-429 in Jurkat cells; whereas it was enhanced at 0~4 h and then inhibited in Hut102 cells (Fig. 5F). Neither in Annexin V/PI detection nor in cell viability detection, Z-VAD-fmk, the pan-caspase inhibitor, inhibited cytotoxicity of FV-429 (Fig. 5G, H). Besides, 25 μM FV-429 had no effect on mitochondrial membrane potential in 0~10 h (Fig. 5I). Unlike rapid loss of mitochondrial membrane potential and caspases cleavage during apoptosis, CICD induces cell death without caspase activation, with a gradual loss of mitochondrial membrane potential, and CICD activation can be inhibited by caspases 8^45,46^. Necroptosis, a programmed CICD, can be suppressed by Necrostatin-1 (Nec-1) via receptor-interacting protein 1 (RIP1) activity inhibition^47,48^. Thus, Nec-1 was used to determine whether caspases mediated FV-429-induced cell death. As shown in Figs. 5G, H and S5B, the cell death rates and cell viability inhibition were inhibited by Nec-1. These data suggested that FV-429 induced CICD in T-cell malignancies.

In addition to the antitumor effects of FV-429 on T-cell malignancies, we also measured the cytotoxicity on normal cells. We found that 25 μM FV-429 only induced a slight cell viability inhibition and cell death of PBMCs derived from healthy donor at 24 h (Fig. 5J, K), suggesting the selective cytotoxicity of FV-429 between normal cells and malignant cells.

### FV-429 sensitized to chemotherapeutic drugs by inhibiting protective autophagy

**Fig. 6:**
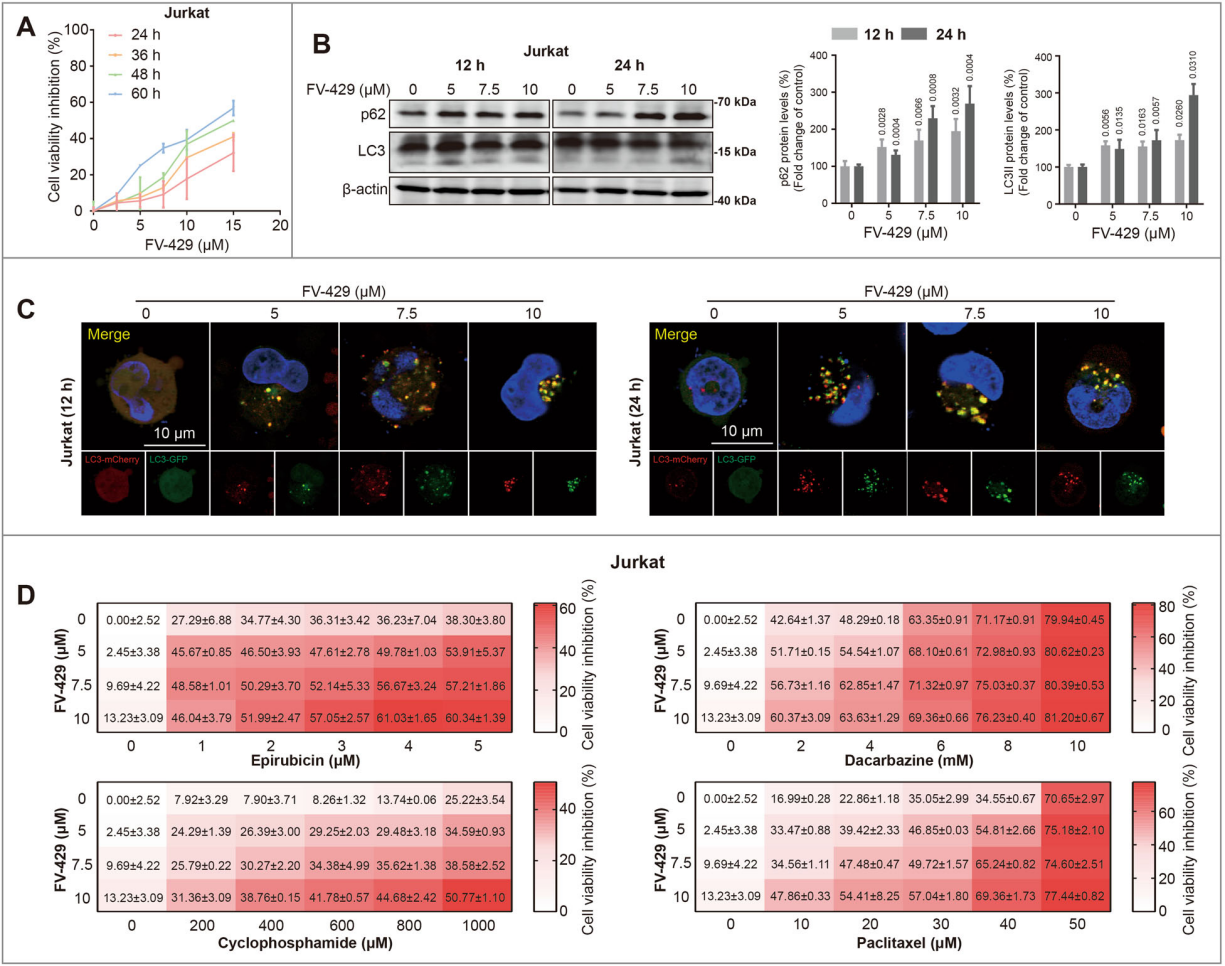
FV-429 sensitizes tumor cells to chemotherapy by inhibiting autophagy. **A** The Jurkat cells were treated with 0~15 μM FV-429 for indicated times, and the cell viability inhibition was determined by CCK8 assay. **B** The Jurkat cells were treated with 0, 5, 7.5, and 10 μM FV-429 for 12 and 24 h, the expression levels of p62 and LC3 were determined by western blot. β-actin used as loading control. The statistically significant differences with respect to 0 μM FV-429 groups. **C** The Jurkat cells transfected with a plasmid encoding LC3-GFP-mcherry were treated with 0, 5, 7.5, and 10 μM FV-429 for 12 and 24 h, and LC3II puncta count were calculated (scale bar: 10 μm; total puncta in each group >200) (mean ± s.e.m. for 3 independent experiments; *p* values are shown on the graph). **D** The Jurkat cells were pre-treated with 0, 5, 7.5, and 10 μM FV-429 for 12 h, and then treated with chemotherapy drugs (Epirubicin, Dacarbazine, Cyclophosphamide, and Paclitaxel) for 24 h. The cell viability inhibition rates (mean ± s.e.m.) were determined by CCK8 assay and shown in heat map.

It has been indicated that autophagy regulates cancer progression and therapy-induced protective autophagy is a way for survival of cancer cells. And in some studies, inhibiting autophagy with chemotherapy treatment is a strategy for eliminating cancer cells by reducing chemotherapy resistance. Therefore, we explored the anticancer efficiency of FV-429 combined with chemotherapy agents. In our experimental principle, the cells were pre-treated with FV-429 to inhibit autophagy flux, and then incubated with chemotherapy agents to determine the cell viability. Although single treatment with 25 μM FV-429 induced cell death significantly, the concentration of FV-429 in combination strategy should not cause notable cell death. First, we screened the concentration of FV-429. As shown in Figs. 6A, 5, 7.5, and 10 μM FV-429 induced a slight cell viability inhibition during 24~48 h in Jurkat cells, with increased expression of LC3II and p62 (Fig. 6B), as well as LC3II puncta emitted yellow fluorescent by LC3-GFP-mCherry plasmid transfection (Fig. 6C). It is suggested that 5, 7.5, and 10 μM FV-429 could inhibit autophagy with low cytotoxicity. Thus, we confirmed the combination efficiency of 5, 7.5, and 10 μM FV-429 with chemotherapy agents in different concentrations. Co-treatment of Jurkat cells with chemotherapy agents and 5, 7.5, and 10 μM FV-429 resulted in decreases of cell viability compared to mono-chemotherapy alone (Fig. 6D). In addition to Jurkat cells, 5, 7.5, and 10 μM FV-429 inhibited autophagy flux in Molt4 cells and promoted cell viability inhibition induced by epirubicin, cyclophosphamide, and paclitaxel, while it hardly inhibited cell viability in Molt4 cells when treated alone (Fig. S6A, B). Superimposed effect was achieved by combination of FV-429 and cyclophosphamide in primary cell #1 (Fig. S6C), and 5 μM FV-429 sensitized epirubicin in primary cell #2 (Fig. S6D). It is demonstrated that the combination of chemotherapy with autophagy inhibition might show significantly more effect on cell viability inhibition to a certain extent.

## Discussion

Autophagy plays an important role in tumor formation and progression as well as with altered response to cancer therapy^49^. Inhibition or promotion of cancer cell growth by autophagy depends on the developmental stage and cell types^50^. Modulating autophagy for cancer therapy is a choice^8^. FV-429, a new derivative of wogonin with bis(2-hydroxyethyl) amino-propoxy substitution, has better water solubility and druggability^51^, and showed superiority of destructive effects on lysosomes than wogonin. We investigated the effect of FV-429 on modulation of autophagy, and indicated that FV-429 blocked autophagy flux and lysosomal degradation pathway by inhibiting lysosome-associated membrane fusion. The autophagy inhibition of FV-429 resulted from targeting lysosomes. The lysosomal dysregulation triggered CICD, which revealed the antitumor efficiency of FV-429 (25 μM) on T-cell malignancies as a single agent.

Due to the reliance of cancer cells on lysosomal function, it makes them more sensitive to lysosome disruption and lysosomotropic agents^52,53^. Recent studies have shown that LMP results in cell death and the impairment of autophagy function, making it possible to treat cancer cells^54,55^. Various agents used alone or in combination with the agents targeting lysosomes show significant anti-tumor effects, and contribute to reduce radiotherapy and chemotherapy resistance in cancer^12,54–56^. In addition, chaperone-mediated autophagy and macroautophagy can be induced as compensatory mechanisms when either mechanism is altered^57^. Targeting lysosomes as a hub of all types of autophagy may counteract their compensation effects^58^. Therefore, development of a new agent to target lysosomes for the treatment of cancer has an important clinical significance. Our results support that FV-429 could enhance cell death in T-cell malignancies as a single agent or in combination. However, we cannot rule out the possibility of the lethal effect of FV-429 on normal cells for long-term treatment, due to the inhibition of cell viability on normal cells induced by 25 μM FV-429 at 36 h (Fig. 5J).

Many factors contribute to permeabilization of the lysosomal membrane^43^. As FV-429 promoted lysosomal enlargement, we speculated that it might be resulting from substances accumulated on lysosomes. Previous studies suggested that the abnormal lysosomal membrane contents of cholesterol or sphingomyelin might result in lysosomal damage^40,59,60^. Accompanied with FV-429-induced lysosomal enlargement, we speculated that the substances accumulated on lysosomes might be resulting from lipid dysregulation, and the regulation of lysosome lipid metabolism would be investigated for further research.

Protective autophagy allows cancer cells to adapt and survive the actions of chemotherapeutic drugs, thus contributing to the further progression of cancers. Many studies have investigated the efficiency of tumor treatment with autophagy inhibition^4,7,61^, or in combination with targeted therapies^62^. In our results of Jurkat cells, FV-429 (5 μM) sensitized to efficiency of chemotherapy drugs with little cytotoxicity (Fig. 6D). However, one point needs to be emphasized: autophagy inhibition is not always beneficial for chemotherapy treatment. For one thing, therapy-induced autophagy can also be cytotoxic^63^; for another thing, inhibition of non-protective autophagy produces barely discernible influence on the therapeutic response^63–65^. At present, the mechanism of autophagy regulation in tumors is still not clear^4^. It is generally accepted that whether autophagy promotes or inhibits cell death depends on the internal and external environment and cell type^3^. We also found the diverse effects of autophagy inhibition on different cells by results of combination treatment in primary cells and cell lines. For example, FV-429 hardly influences the cell viability in Molt4 cells treated with dacarbazine, suggesting that the autophagy inhibition had no effect upon treatment with dacarbazine (Fig. S6B). As for primary cells #1, although combination of FV-429 and cyclophosphamide achieved higher cell viability inhibition than FV-429 or cyclophosphamide-treated alone, it was more like the superimposed effect. Besides, FV-429 hardly affected the cell viability inhibition induced by epirubicin, dacarbazine, and paclitaxel (Fig. S6C). In primary cells #2, only the efficiency of epirubicin treatment was improved by FV-429 (Fig. S6D). These results indicated that the effects of autophagy inhibition on chemotherapeutic sensitization was not absolute. However, T-cell malignancies encompass a heterogeneous group of diseases. There were only two kinds of primary cells that were used in combination treatment due to the limitation of primary cells. Therefore, the conclusion should be proved in more types of T-cell malignancies and even performed in vivo.

Previous studies have shown that the examples of calcium-regulated autophagy vesicle formation, which can be inhibited by BAPTA-AM in human neuroblastoma cell line SH-SY5Y, HEK 293T, and duck embryo fibroblast cells and so on^66–69^. In contrast to Burkitt’s lymphoma cell lines Raji, BAPTA-AM increased LC3II expression when treated alone^70^. We verified that BAPTA-AM inhibited autophagy vesicle formation in Jurkat and Hut102 cells treated with FV-429 (Figs. 1E and S1B). However, in Molt4 cells, BAPTA-AM promoted FV-429-induced LC3II expression significantly (Fig. S1D). As far as we all know, we can only extrapolate that the differences of cytoplasmic calcium regulation on autophagy might be due to the diversity of T-cell malignancies.

In summary, we demonstrated that FV-429 inhibited autophagy in T-cell malignancies via inducing lysosomal damage and promoted autophagy vesicle accumulation and LCD. These findings revealed the antitumor effects of FV-429 on T-cell malignancies, and targeting lysosomes or autophagy inhibition might be a potential therapeutic strategy for the tumor treatment.

## Supplementary information

Supplemental Fig. 1.

Supplemental Fig. 2.

Supplemental Fig. 3.

Supplemental Fig. 4.

Supplemental Fig. 5.

Supplemental Fig. 6.

Supplementary Figure legend.

## Data Availability

All data generated or analyzed during this study are included in this published article.
