# Supplementary figures and images for: FV-429 induces autophagy blockage and lysosome-dependent cell death of T-cell malignancies via lysosomal dysregulation
Source: Cell Death Dis. 2021 Jan 13;12(1):80. doi: 10.1038/s41419-021-03394-4 (PMC7806986; doi:10.1038/s41419-021-03394-4)

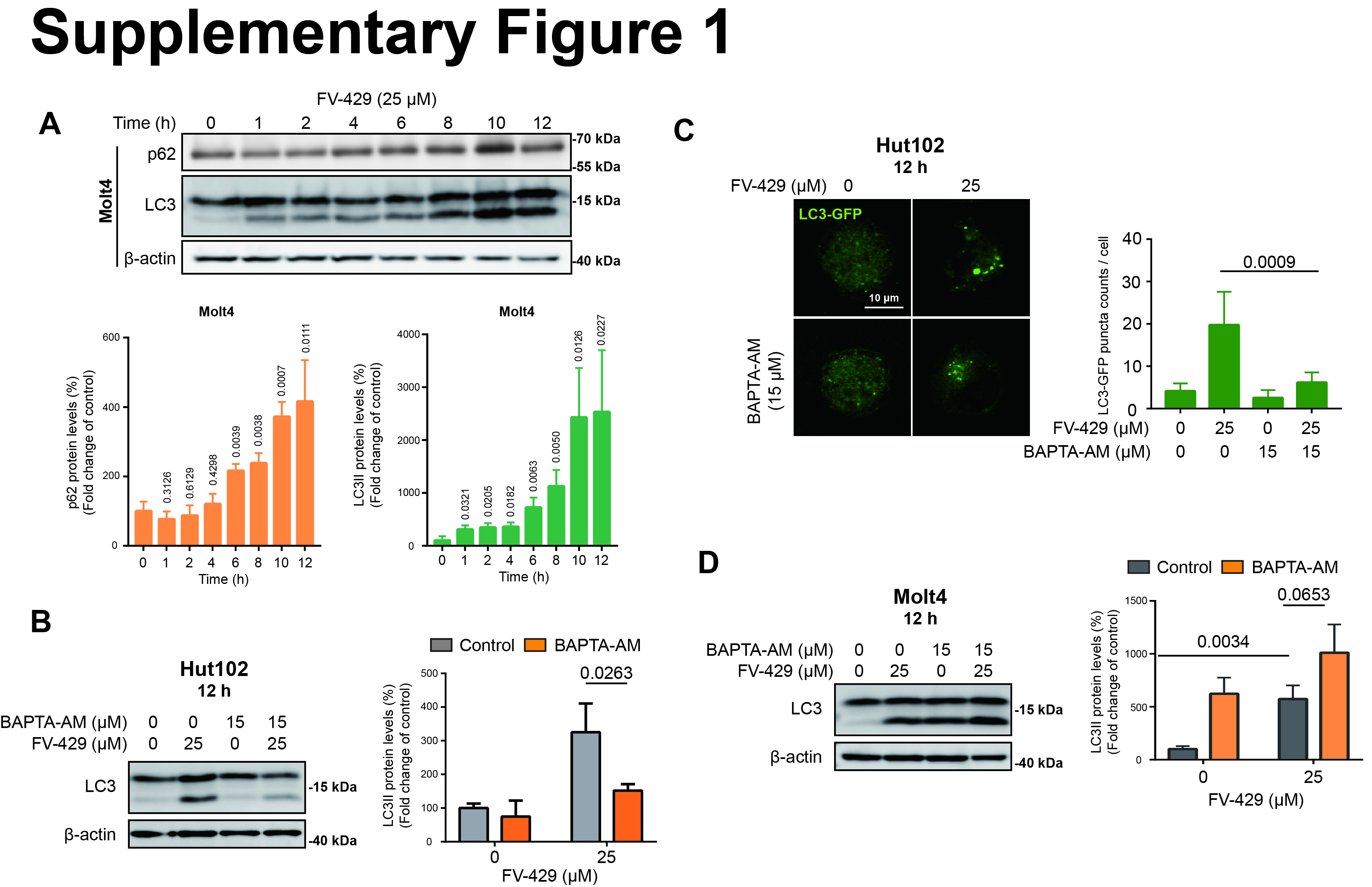

Supplement: Supplementary file 1 — Supplemental Fig. 1. [file 41419_2021_3394_MOESM1_ESM.jpg]

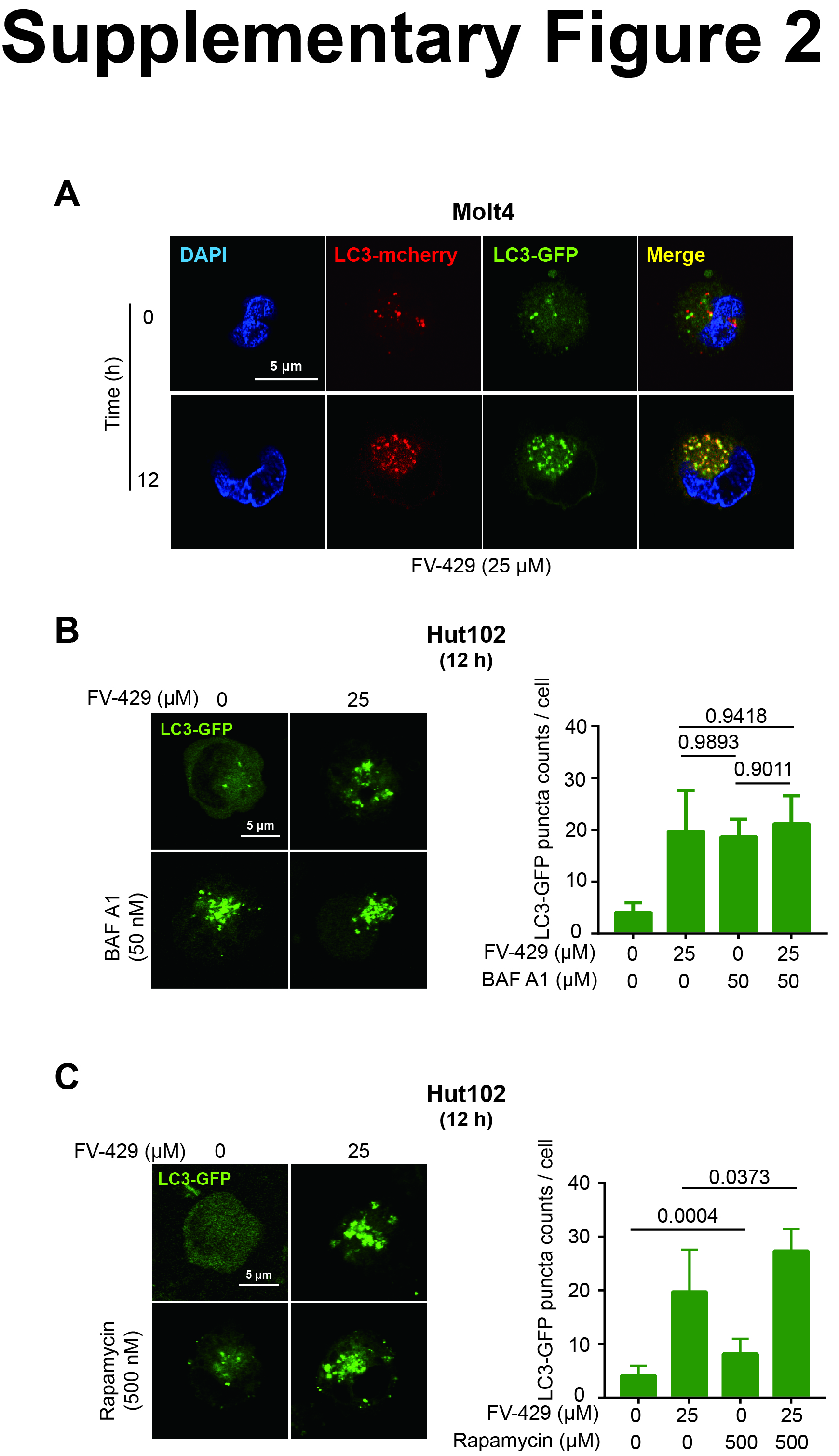

Supplement: Supplementary file 2 — Supplemental Fig. 2. [file 41419_2021_3394_MOESM2_ESM.jpg]

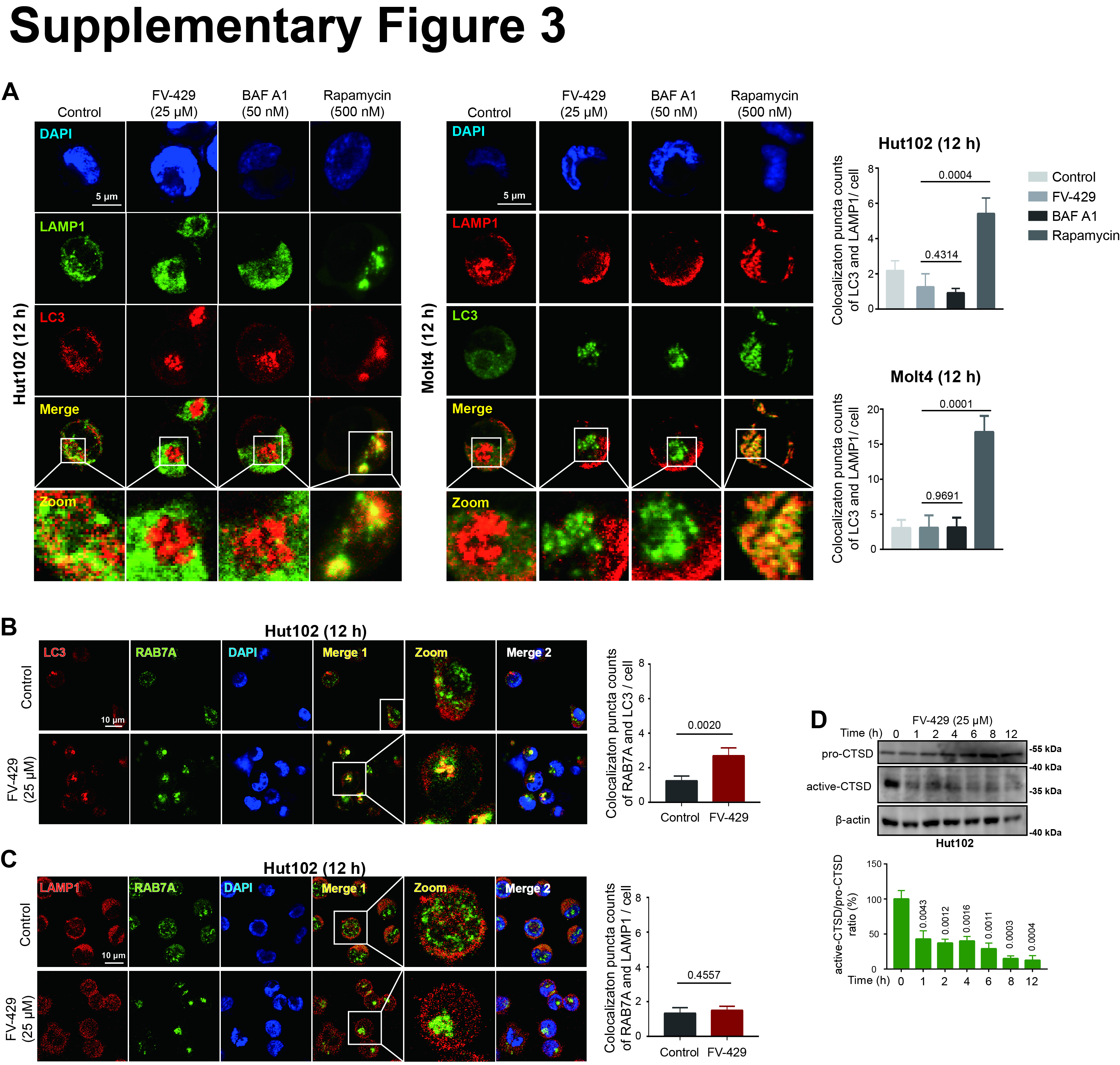

Supplement: Supplementary file 3 — Supplemental Fig. 3. [file 41419_2021_3394_MOESM3_ESM.jpg]

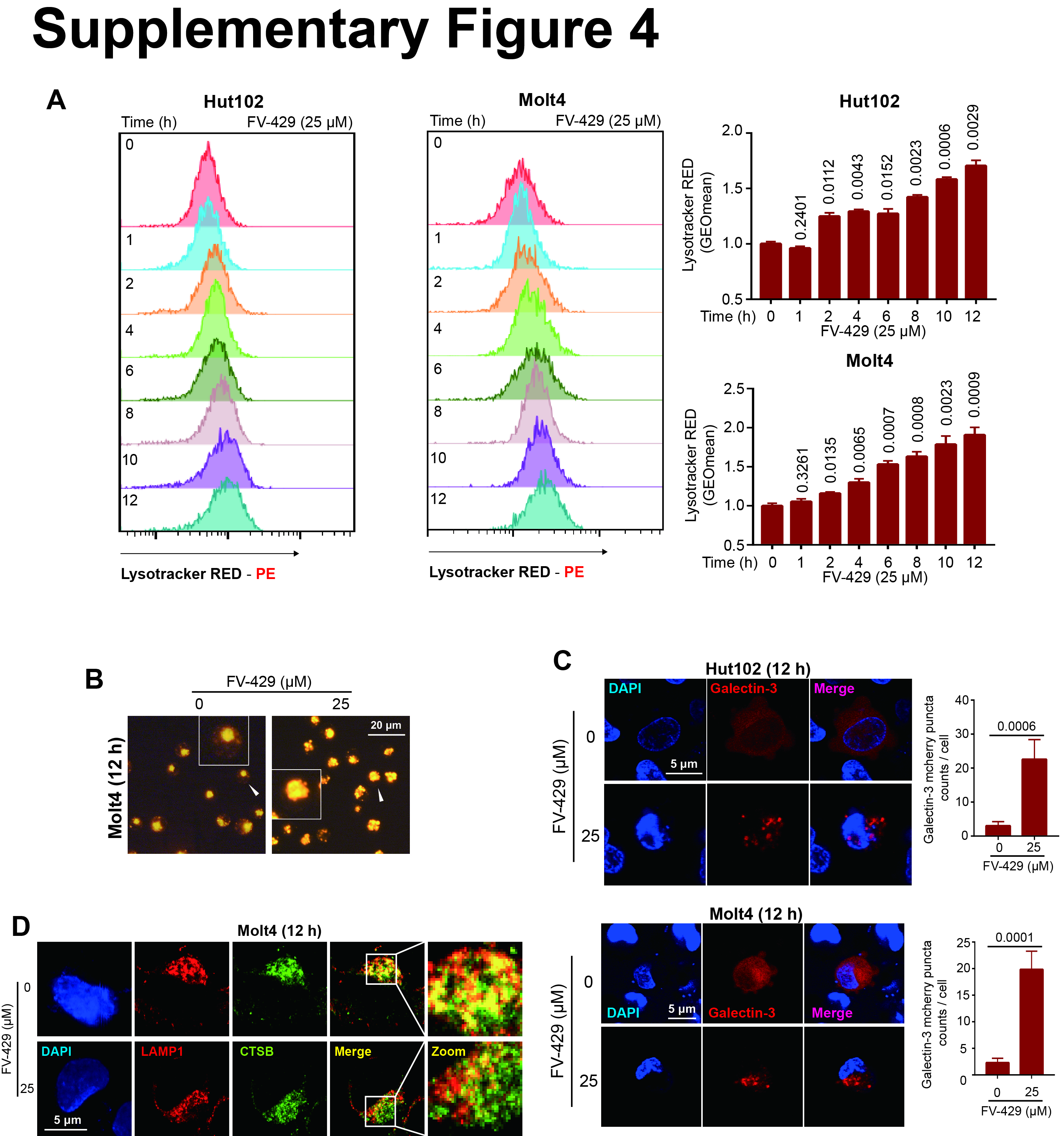

Supplement: Supplementary file 4 — Supplemental Fig. 4. [file 41419_2021_3394_MOESM4_ESM.jpg]

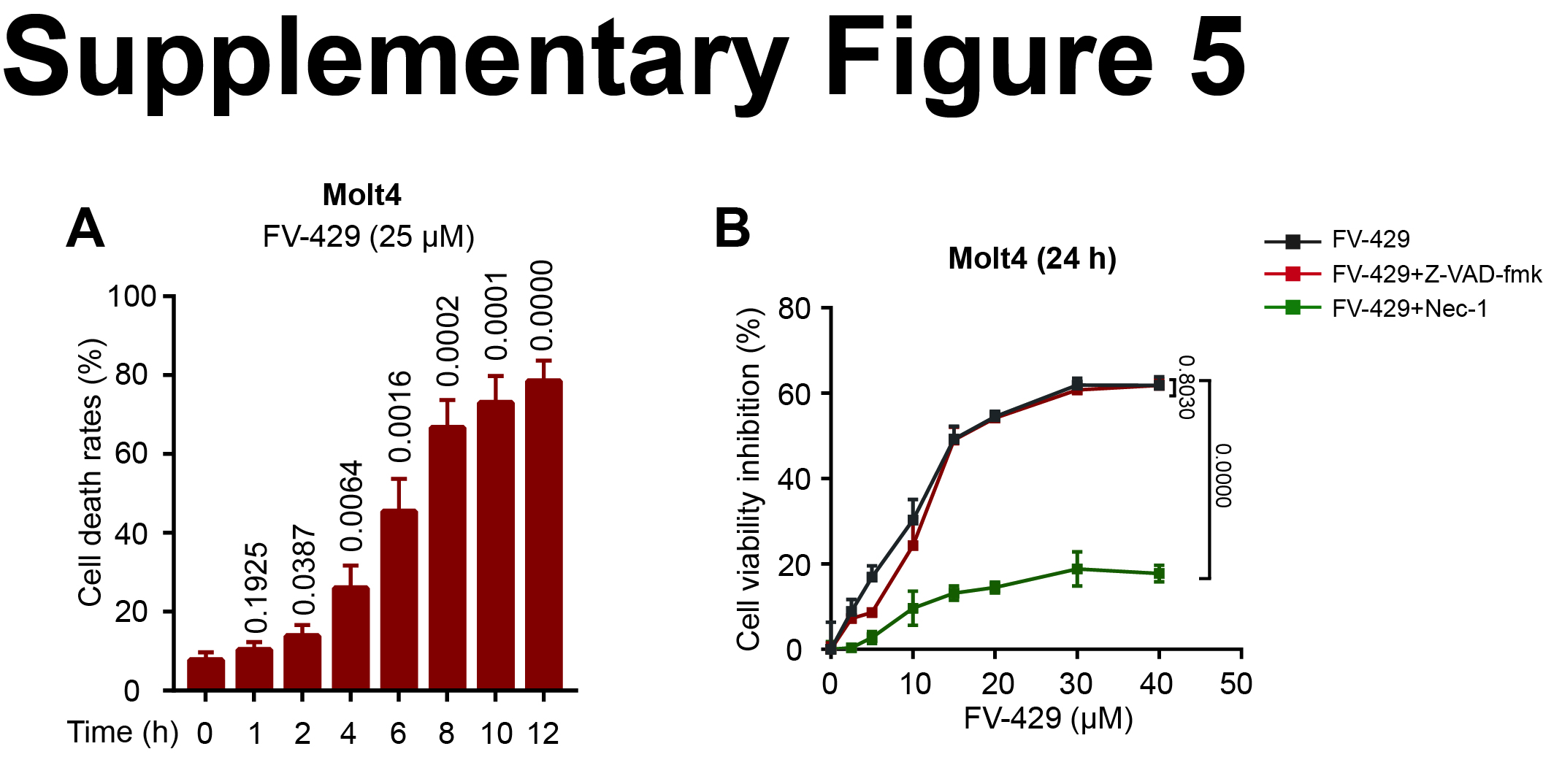

Supplement: Supplementary file 5 — Supplemental Fig. 5. [file 41419_2021_3394_MOESM5_ESM.jpg]

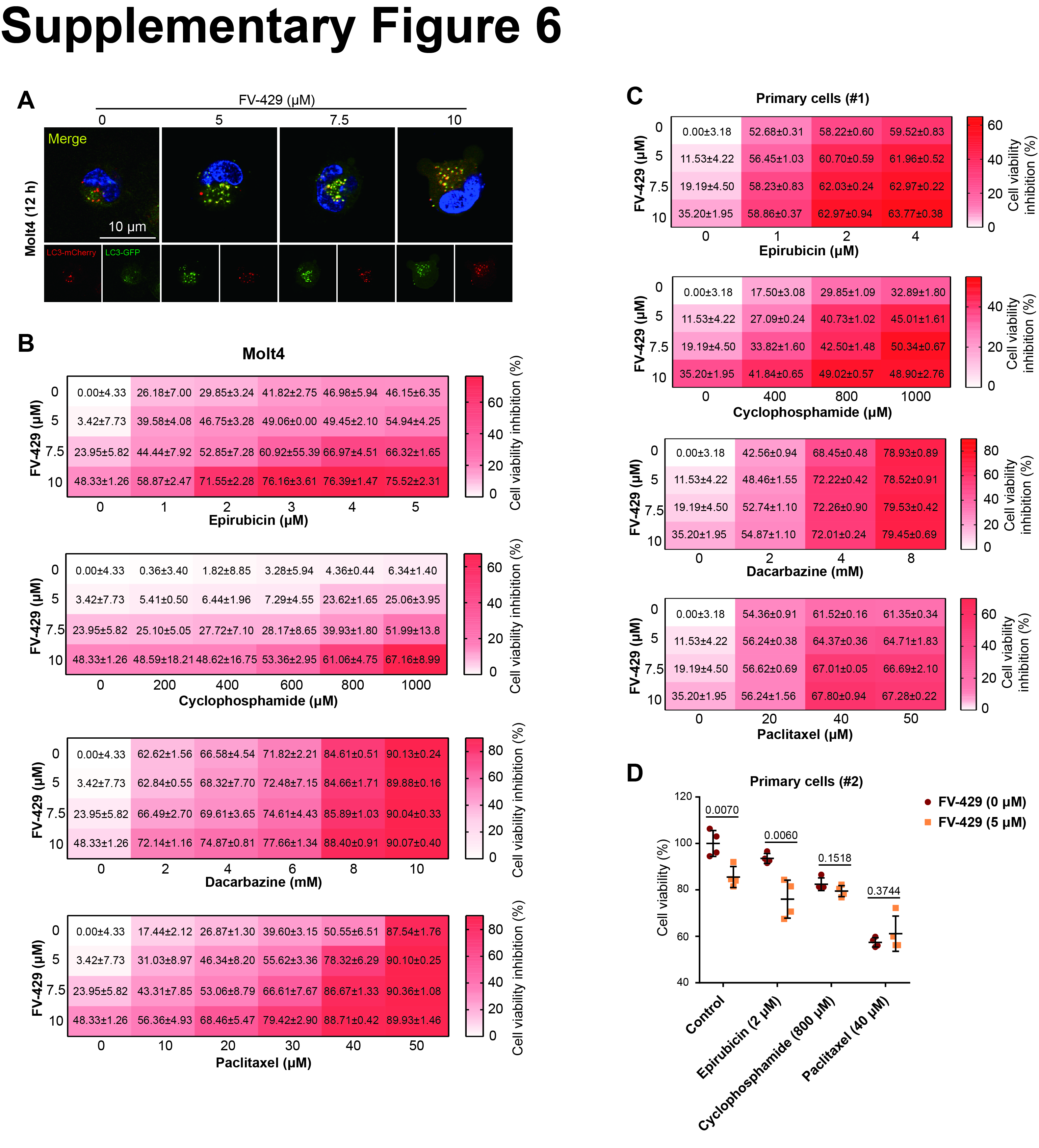

Supplement: Supplementary file 6 — Supplemental Fig. 6. [file 41419_2021_3394_MOESM6_ESM.jpg]
