# Supplementary material for: FV-429 induces autophagy blockage and lysosome-dependent cell death of T-cell malignancies via lysosomal dysregulation
Source: Cell Death Dis. 2021 Jan 13;12(1):80. doi: 10.1038/s41419-021-03394-4 (PMC7806986; doi:10.1038/s41419-021-03394-4)
Supplement: Supplementary file 7 — Supplementary Figure legend. [file 41419_2021_3394_MOESM7_ESM.docx]

**Supplementary Figure legends**

**Supplementary Figure 1**

(A) The Molt4 cell were treated with 25 μM FV-429 for 0~12 h, and the expression of p62 and LC3 were determined by western blot. β-actin as loading control in western blot.

(B) The Hut102 cells were treated with 25 μM FV-429 and BAPTA-AM (15 μM, pre-treated for 1 h) for 12 h, the protein expression of LC3 was determined by western blot. β-actin as loading control in western blot.

(C) The Jurkat cells were transfected with a plasmid encoding LC3-GFP and treated with 25 μM FV-429 and BAPTA-AM (15 μM, pre-treated for 1 h) for 12 h. The LC3Ⅱ puncta determined by fluorescence microscopy, and LC3Ⅱ-GFP puncta counts were calculated (scale bar: 10 μm; total puncta in each group > 200).

(D) The Molt4 cells were treated with 25 μM FV-429 and BAPTA-AM (15 μM, pre-treated for 1 h) for 12 h, the protein expression of LC3 was determined by western blot. β-actin as loading control in western blot. (mean ± s.e.m. for 3 independent experiments; *p* values are shown on the graph)

**Supplementary Figure 2**

(A) The cells transfected with a plasmid encoding LC3-GFP-mcherry were incubated with 25 μM FV-429 for 12 h in Molt4 cells. The LC3Ⅱ puncta formation was detected by fluorescence microscopy (scale bar: 5 μm).

(B) The Hut102 cells were transfected with a plasmid encoding LC3-GFP and treated with 25 μM FV-429 and BAF A1 (50 nM, pre-treated for 1 h) for 12 h. The LC3Ⅱ puncta determined by fluorescence microscopy, and LC3Ⅱ-GFP puncta counts were calculated (scale bar: 5 μm; total puncta in each group > 200).

(C) The Hut102 cells were transfected with a plasmid encoding LC3-GFP and treated with 25 μM FV-429 and rapamycin (500 nM, pre-treated for 2 h) for 12 h. The LC3Ⅱ puncta determined by fluorescence microscopy, and LC3Ⅱ-GFP puncta counts were calculated (scale bar: 5 μm; total puncta in each group > 200). (mean ± s.e.m. for 3 independent experiments; *p* values are shown on the graph)

**Supplementary Figure 3**

(A) The Hut102 and Molt4 cells were treated with FV-429, BAF A1 and rapamycin for 12 h. The immunofluorescence analysis performed with anti-LAMP1 antibody, anti-LC3 antibody and DAPI. The overlay levels were analyzed (scale bar: 5 μm, the cells calculated in each group > 100).

(B-C) The Hut102 cells were treated with 25 μM FV-429 for 12 h. The immunofluorescence analysis performed with anti-LC3 antibody (red), anti-RAB7A antibody (green) and DAPI (blue; nuclei) (B); or performed with anti-LAMP1 antibody (red), anti-RAB7A antibody (green) and DAPI (blue; nuclei) (C). The co-localization counts of RAB7A with LC3 or LAMP1 were calculated (scale bar: 10 μm, the cells calculated in each group > 100).

(D) The Hut102 cells were treated with 25 μM FV-429 for 0~12 h, and the expression of CTSD were determined by western blot. β-actin as loading control (mean ± s.e.m. for 3 independent experiments; *p* values are shown on the graph).

**Supplementary Figure 4**

(A) The Hut102 and Molt4 cells treated with 25 μM FV-429 for 0~12 h were stained by Lysotracker RED and detected by flow cytometry. GEOmean of fluorescence intensity were analyzed by FlowJo software.

(B) The Molt4 cells were treated with 25 μM FV-429 for 12 h and stained with Lysotracker RED. The morphology was determined by fluorescent microscope (scale bar: 20 μm).

(C) The Hut102 and Molt4 cells transfected with Galectin-3 mcherry plasmid were treated with 25 μM FV-429 for 12 h and the Galectin-3 puncta were calculated (scale bar: 5 μm; total puncta in each group > 100) (mean ± s.e.m. for 3 independent experiments; *p* values are shown on the graph).

(D) The Molt4 cells were treated with 25 μM FV-429 for 12 h. The immunofluorescence analysis performed with anti-LAMP1 antibody (red; lysosomes), anti-CTSB antibody (green) and DAPI (blue; nuclei) (scale bar: 5 μm)

**Supplementary Figure 5**

(A) The Molt4 cells were treated with 25 μM FV-429 for 0~12 h. The cells were stained with Annexin V/PI staining and cell death rates were detected by flow cytometry (death cells are positive for Annexin V).

(B) The Molt4 cells were pre-treated with Z-VAD-fmk (15 μM) or Nec-1 (100 μM) for 2 h and then incubated with 0~40 μM FV-429 for 24 h. The cell viability inhibition was determined by CCK8 assay (mean ± s.e.m. for 3 independent experiments; *p* values are shown on the graph).

**Supplementary Figure 6**

(A) The Molt4 cells transfected with a plasmid encoding LC3-GFP-mcherry were treated with 0, 5, 7.5 and 10 μM FV-429 for 12 h. and LC3Ⅱ puncta counts were calculated (scale bar: 10 μm; total puncta in each group > 100) (mean ± s.e.m. for 3 independent experiments; *p* values are shown on the graph).

(B) The Jurkat cells were pre-treated with 0, 5, 7.5 and 10 μM FV-429 for 12 h, and then treated with chemotherapy drugs (Epirubicin, Dacarbazine, Cyclophosphamide and Paclitaxel) for 24 h. The cell viability inhibition rates (mean ± s.e.m.) were determined by CCK8 assay and shown in heat-map.

(C) The primary cells #1 (mature T cell leukemia) were pre-treated with 0, 5, 7.5 and 10 μM FV-429 for 12 h, and then treated with chemotherapy drugs (Epirubicin, Dacarbazine, Cyclophosphamide and Paclitaxel) for 24 h. The cell viability inhibition rates (mean ± s.e.m.) were determined by CCK8 assay and shown in heat-map.

(D) The primary cells #2 (immature T-ALL) were pre-treated with 0, and 5 μM FV-429 for 12 h, and then treated with chemotherapy drugs (Epirubicin, Cyclophosphamide and Paclitaxel) for 24 h. The cell viability inhibition rates (mean ± s.e.m.) were determined by CCK8 assay.
